# Different Kynurenine Pathway Dysregulation in Systemic Sclerosis in Men and Women

**DOI:** 10.3390/ijms25073842

**Published:** 2024-03-29

**Authors:** Monika Turska-Kozłowska, Bruno Pedraz-Petrozzi, Piotr Paluszkiewicz, Jolanta Parada-Turska

**Affiliations:** 1 Department of Molecular Biology, The John Paul II Catholic University of Lublin, Konstantynow 1H, 20-708 Lublin, Poland; 2 Department of Psychiatry and Psychotherapy, Central Institute of Mental Health, Medical Faculty Mannheim, University of Heidelberg, J5, 68159 Mannheim, Germany; bruno.pedrazpetrozzi@zi-mannheim.de; 3 Department of General, Oncological and Metabolic Surgery, Institute of Hematology and Transfusion Medicine, Indiry Gandhi 14, 02-778 Warsaw, Poland; ppaluszkiewicz@ihit.waw.pl; 4 Department of Rheumatology and Connective Tissue Diseases, Medical University of Lublin, Jaczewskiego 8, 20-090 Lublin, Poland; jolanta.parada-turska@umlub.pl

**Keywords:** tryptophan, kynurenine, kynurenic acid, kynurenine pathway, scleroderma

## Abstract

Systemic sclerosis (SSc), a predominantly female-affected systemic autoimmune disease, requires tailored treatment strategies contingent on organ involvement and symptom severity. Given SSc’s inflammatory nature, the involvement of the kynurenine pathway (KP) in its pathophysiology is underexplored. Our study aimed to investigate sex-related differences in KP activation among SSc patients and assess the impact of angiotensin-converting enzyme (ACE) inhibitors and estimated glomerular filtration rate (eGFR) on KP metabolite concentrations. We enrolled 48 SSc patients and 53 healthy controls, quantifying KP metabolites (tryptophan (TRP), kynurenine (KYN), and kynurenic acid (KYNA)) in serum via high-performance liquid chromatography. Separate multivariate analyses of covariance (MANCOVAs) for women and men were performed to ascertain mean differences between patients and healthy controls while correcting for age. For our secondary objective, we conducted a MANCOVA to explore disparities in ACE inhibitor users and non-users among patients, with BMI correction. Our findings revealed decreased TRP concentrations but increased KYNA/TRP ratio and KYN/TRP ratio in both male and female SSc patients compared to their respective controls. Unlike women, SSc males exhibited higher KYN concentrations and decreased KYNA/KYN ratio relative to their controls. Additionally, SSc patients using ACE inhibitors had higher serum KYNA levels than non-users. Notably, we established a significant correlation between eGFR and KYNA in SSc patients. These results indicate differential KP activation in male and female SSc patients, with males demonstrating heightened KP activation. While ACE inhibitors may influence the KP in SSc patients, further research is necessary to comprehensively understand their impact on symptoms and prognosis in the context of these KP alterations.

## 1. Introduction

Systemic sclerosis (SSc) is an autoimmune connective tissue disease, characterized by vascular abnormalities and fibrosis, resulting in atrophic processes in skin and organ tissues (i.e., in gastrointestinal tract, in lower respiratory tract including lungs, in cardiac muscle, and in kidneys). Worldwide it is estimated that SSc has a prevalence of 17.6 per 100,000 persons, affecting more women than men (5:1) [1,2].

Although the causes of SSc remain unclear, a range of genetic, immunological, and environmental factors [2] are hypothesized to be the primary contributors. It has been proposed, however, that an early alteration in the vascular endothelium leads to the eventual development of systemic, extensive fibrosis, serving as the overarching pathophysiological mechanism [2]. The latter serves also as a classification criterion for SSc, besides the presence of characteristic antibodies, such as anti-centromere (ACA), anti-topoisomerase I (Scl-70), and anti-RNA polymerase III [3]. As the cause remains unclear, there are currently no identified pharmacological targets, and consequently, effective treatments for the disease are lacking. Recommendations for treatment primarily focus on symptom reduction and the mitigation of organ dysfunction. For instance, in the case of renal impairment, angiotensin-converting enzyme (ACE) inhibitors are commonly employed in SSc.

**Scheme 1:**
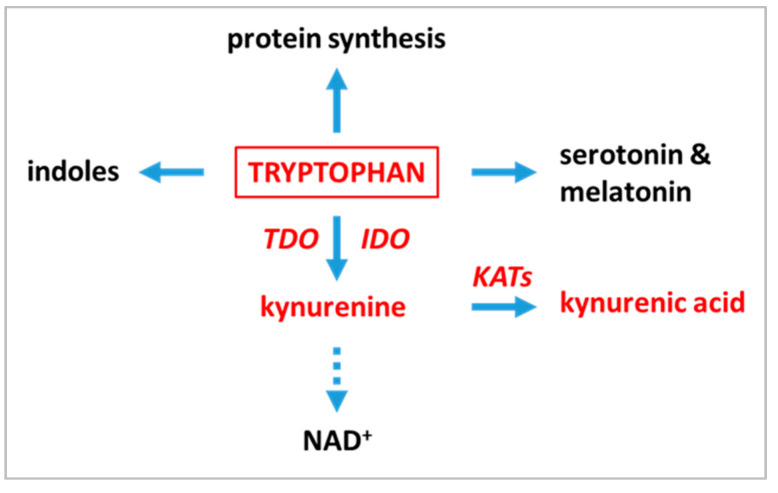
Simplified scheme of tryptophan metabolism in mammals. Note that only the main products are marked on the diagram. The compounds and enzymes analyzed in the publication are shown in red. IDO–indoleamine 2,3-dioxygenase; KATs—kynurenine aminotransferases; TDO–tryptophan 2,3-dioxygenase.

Tryptophan (TRP), one of the nine essential amino acids for humans, is used not only for protein synthesis, but undergoes transformations through several metabolic pathways. In the gastrointestinal tract the microbiome mainly produces indoles, while absorbed tryptophan is metabolized in tissues along the kynurenine and serotonin pathways of which the kynurenine pathway (KP) represents a major route of metabolism of TRP (Scheme 1) [4].

In recent decades, there has been increasing discussion about the role of KP metabolites in SSc [5,6,7], as these metabolites are closely associated with inflammation [8]. Previous studies in SSc patients have shown an increase in the urinary excretion of certain TRP metabolites, particularly kynurenine (KYN), which allowed formulation of the hypothesis regarding dysregulation of TRP metabolism in SSc [9,10,11,12]. Furthermore, other studies revealed that metabolites of TRP formed along KP, specifically KYN and kynurenic acid (KYNA), have been found to inhibit fibroblast metabolism and proliferation and alleviate fibrosis [13,14]. Additionally, phase I/II clinical studies have demonstrated the use of KYNA as an anti-scarring medication, demonstrating its potential clinical utility [15,16].

The role of serotonin in SSc, mainly as a mediator of fibrosis and vasculopathy, has been recently reviewed [17].

Despite the growing body of literature indicating progress in comprehending the KP in SSc patients, studies addressing sex-related differences in KP activation are insufficient. Such differences are crucial in the context of SSc, as the condition tends to manifest more severely with a higher incidence of complications and a graver prognosis in men as compared to women [18,19]. Currently, there is a growing interest in gender-dependent phenomena in SSc, and the need to take them into consideration in both the diagnosis and treatment process has been postulated [20,21].

To this end, the main aim of this study is to examine the sex-related differences in the KP in SSc patients considering the peripheral concentrations of TRP, KYNA, and KYN as well as kynurenine pathway enzyme activity indicators, indoleamine 2,3-dioxygenase (IDO) and tryptophan 2-3dioxygenase (TDO), represented by the ratio of KYN/TRP and kynurenine aminotransferases (KATs) calculated as the KYNA/KYN ratio and KYNA/TRP ratio (see Scheme 1), while controlling for age. As a secondary objective, we aim to investigate the influence of ACE inhibitors and estimated glomerular filtration rate (eGFR) on concentration of TRP, KYNA, and KYN. To achieve this, we will consider BMI as covariate.

## 2. Results

### 2.1. General Characteristics of the Sample

General characteristics of both participant groups (patients and healthy controls) are presented in Table 1. Regarding patient characteristics, the patient group had a mean BMI of 23.40 ± 5.23 kg/m^2^. The mean age of onset for scleroderma was 46.13 ± 11.98 years, and the mean disease duration since the onset was 6.25 ± 5.23 years. Concerning disease-related antibodies, 75% of participants with scleroderma (36/48) tested positive for Scl-70 antibodies, 8.33% (4/48) had positive anti-ACA antibodies, 10.42% (5/48) had anti-PM/Scl antibodies, 14.58% (7/48) had anti-SS-A antibodies, 10.42% (5/48) had anti-SS-B antibodies, 6.25% (3/48) had anti-Jo-1 antibodies, one participant had anti-Mi-2 antibodies, one participant had anti-Sm antibodies, one participant had anti-AMA-M2 antibodies, and one participant had anti-RNP/Sm antibodies. None of the tested antibodies appeared in 12.50% (6/48) of SSc patients.

Regarding disease manifestation, 31.25% (15/48) of participants with systemic scleroderma presented with a limited clinical form (lSSc), while 68.75% (33/48) presented with a diffuse form (dSSc). Among the participants, 97.92% (47/48) had Raynaud syndrome, 89.58% (43/48) had interstitial lung disease, 60.42% (29/48) had telangiectasia, 52.08% (25/48) had fingertip scars, 47.92% (23/48) had esophageal involvement, 35.42% (17/48) had arterial hypertension, 33.33% (16/48) had myositis, 29.17% (14/48) had joint involvement, 29.17% (14/48) had ulcers, 22.92% (11/48) had arrhythmias, 18.75% (9/48) had heart disease, and 2.08% (1/48) had pulmonary hypertension. Raynaud’s sign is a very common symptom found in SSc, which, particularly in lSSc, can precede other disease symptoms by up to several years. In lSSc, vascular manifestations such as Raynaud’s phenomenon, finger ulcers, and telangiectasias often dominate the clinical presentation from the onset of the disease, whereas organ manifestations, including interstitial lung disease, dysphagia, and pulmonary arterial hypertension appear in later stages of the disease. In patients with dSSc, the onset of the illness is usually more severe. Skin lesions and vascular manifestations appear early and almost simultaneously. Furthermore, dangerous organ complications such as interstitial lung disease, gastrointestinal complications, myocardial involvement, and scleroderma renal breakthrough occur already in the first years of the disease [22,23].

Regarding renal involvement, the mean serum creatinine concentration was 0.81 ± 0.19 mg/dL, and the mean estimated glomerular filtration rate (eGFR) was 83.33 ± 11.64 mL/min/1.73 m^2^. Among the participants, 39.58% (19/48) took ACE inhibitors as an antihypertensive agent at the time of the study.

### 2.2. Tryptophan and Its Metabolites—Differences between Male Scleroderma Patients and Male Healthy Controls

TRP and the combined TRP breakdown metabolites (KYN and KYNA), enzyme activity indicators (KYN/TRP equivalent of IDO and TDO activity, KYNA/KYN equivalent of KATs, and KYNA/TRP ratio), measured in male SSc patients and male healthy controls, were analyzed using MANCOVA. In this case, the grouping factor was the status (“patient” and “healthy control”). Moreover, the variable age was considered as a covariate (Table 2). Regarding the MANCOVA multivariate tests, our results indicate a statistically significant interaction with status on the combined dependent variables after controlling for the variable age (F_6,38_ = 11.701, Pillai’s trace = 0.649, *p* < 0.001). In addition, univariate tests showed differences between male SSc and HC participants in the interaction *status* for the TRP, KYN, IDO and TDO, KYNA/TRP, and KATs (TRP: F_1,43_ = 13.715, *p* = 0.001, η^2^_p_ = 0.242; KYN: F_1,43_ = 19.637, *p* < 0.001, η^2^_p_ = 0.314; IDO and TDO: F_1,43_ = 50.391, *p* < 0.001, η^2^_p_ = 0.540; KYNA/TRP: F_1,43_ = 6.418, *p* = 0.015, η^2^_p_ = 0.130; KATs: F_1,43_ = 9.512, *p* = 0.004, η^2^_p_ = 0.181). More details of the MANCOVA results, multivariate and univariate tests, are reported in Table 2.

**Figure 1:**
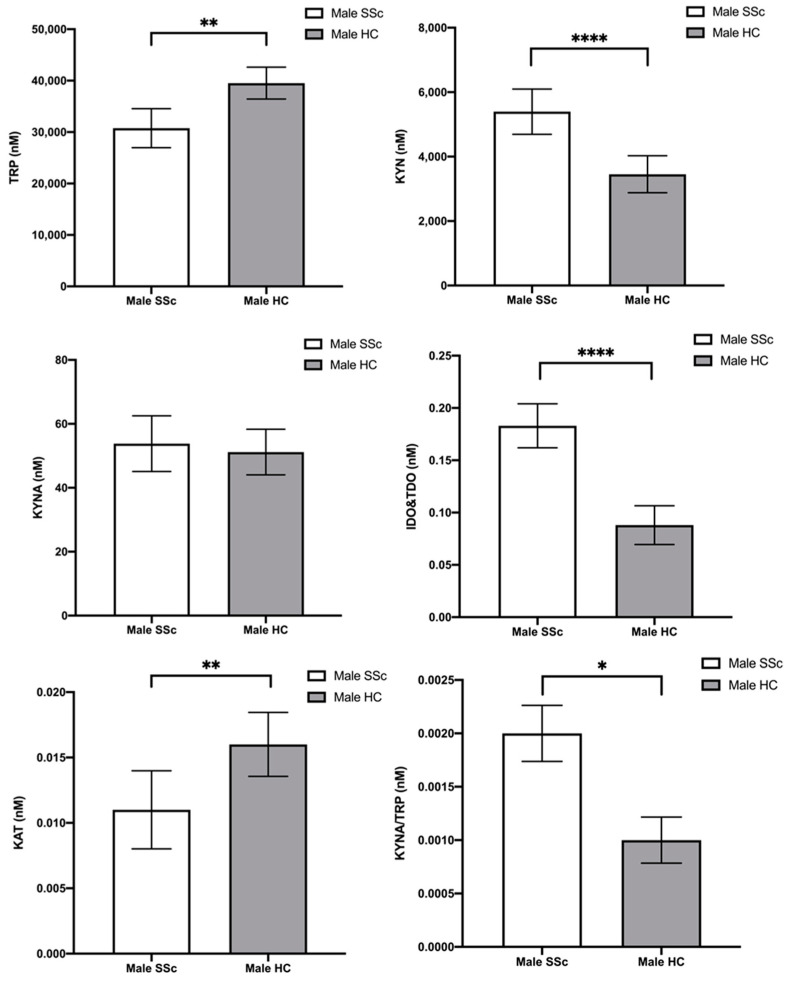
Plasma content of tryptophan (TRP), kynurenine (KYN), and kynurenic acid (KYNA) and enzyme activity indicator in male scleroderma patients (SSc) and male controls (HC). Statistical analysis was performed using Bonferroni corrected post hoc test; * *p* ≤ 0.05, ** *p* ≤ 0.01, **** *p* ≤ 0.0001.

Finally, post hoc comparisons using the Bonferroni *t*-test correction indicated that male SSc patients had: (1) lower TRP concentrations than male HC (M_D_ = −8767.50; *p* = 0.001; 95%CI [−13541.948; −3993.059]), (2) higher KYN concentrations than male HC (M_D_ = 1942.713; *p* < 0.001; 95%CI [1058.594; 2826.832]), (3) higher IDO and TDO activity than male HC (M_D_ = 0.095; *p* < 0.001; 95%CI [0.068; 0.122]), (4) lower KAT activity than male HC (M_D_ = −0.006; *p* = 0.004; 95%CI [−0.010; −0.002]), and (5) higher KYNA/TRP ratio than male HC (M_D_ = 0.001; *p* = 0.015; 95%CI [8.502 × 10^−5^; 0.001]). The remaining post hoc comparisons did not show significant differences. Post hoc analyses are graphically represented in Figure 1.

### 2.3. Tryptophan and Its Metabolites—Differences between Female Scleroderma Patients and Female Healthy Controls

Likewise, we performed a similar analysis with all female participants (female SSc patients and female HC). Regarding the MANCOVA multivariate tests, our results indicate a statistically significant interaction with status on the combined dependent variables after controlling for the variable age (F_6,47_ = 5.707, Pillai’s trace = 0.421, *p* < 0.001). In addition, univariate tests showed differences between female SSc and HC participants in the interaction status for the TRP, IDO and TDO, and KYNA/TRP (TRP: F_1,52_ = 19.414, *p* < 0.001, η^2^_p_ = 0.272; IDO and TDO: F_1,52_ = 14.284, *p* < 0.001, η^2^_p_ = 0.215; KYNA/TRP: F_1,52_ = 11.420, *p* = 0.001, η^2^_p_ = 0.180). More details of the MANCOVA results, multivariate and univariate tests are reported in Table 3.

**Figure 2:**
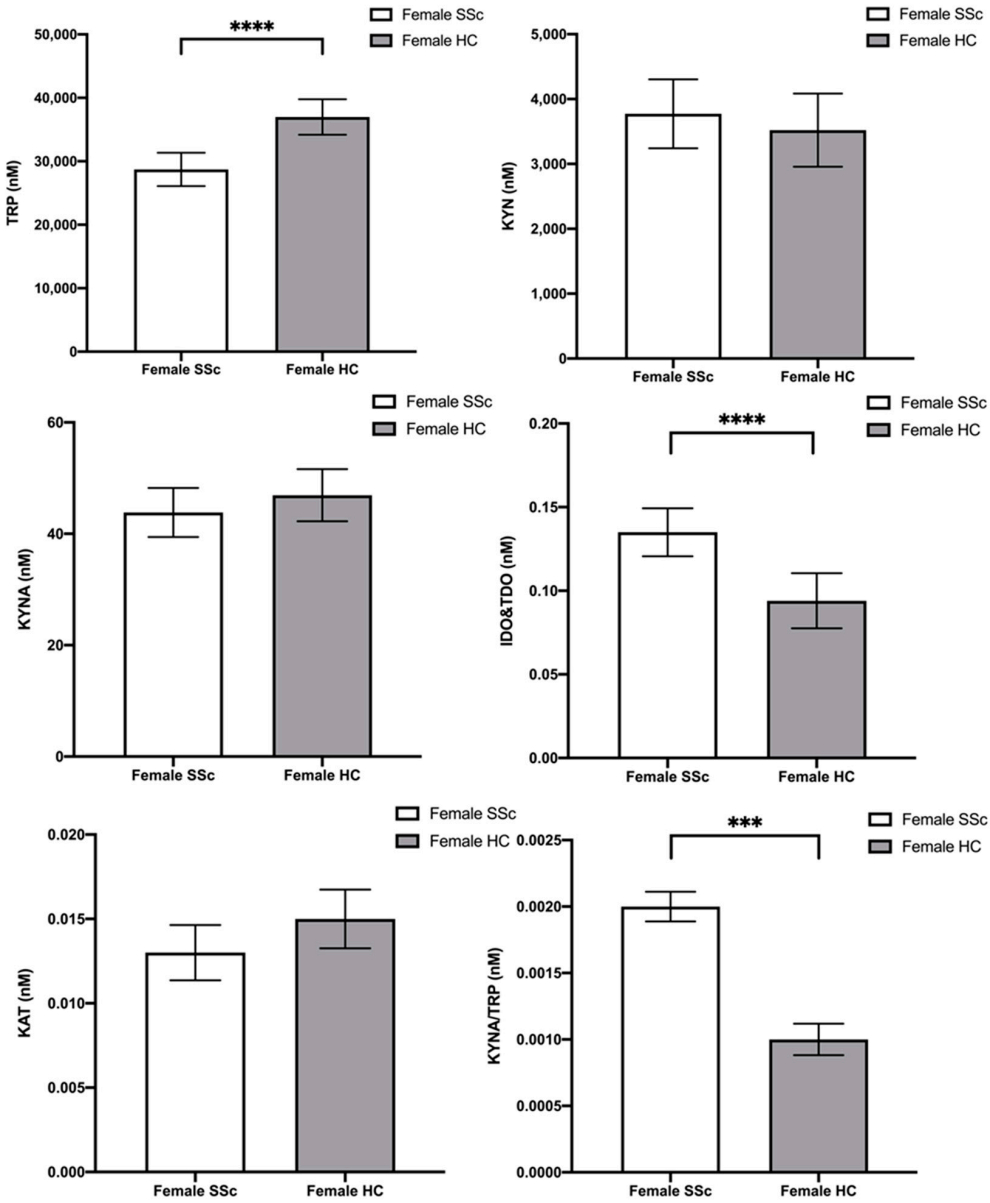
Plasma content of tryptophan (TRP), kynurenine (KYN), and kynurenic acid (KYNA) and enzyme activity indicator in female scleroderma patients (SSc) and female controls (HC). Statistical analysis was performed using Bonferroni corrected post hoc test; *** *p* ≤ 0.001, **** *p* ≤ 0.0001.

Finally, post hoc comparisons using the Bonferroni *t*-test correction indicated that female SSc patients had: (1) lower TRP concentrations than female HC (M_D_ = −8264.430; *p* < 0.001; 95%CI [−12,028.183; −4500.678]), (2) higher IDO and TDO activity than female HC (M_D_ = 0.041; *p* < 0.001; 95%CI [0.019; 0.062]), and (3) higher KYNA/TRP activity than female HC (M_D_ = 0.001; *p* = 0.001; 95%CI [1.09 × 10^−4^; 4.29 × 10^−4^]). The remaining post hoc comparisons did not show significant differences. Post hoc analyses are graphically represented in Figure 2.

### 2.4. Tryptophan and Its Metabolites—Effects of the eGFR and Use of ACE Inhibitors

**Figure 3:**
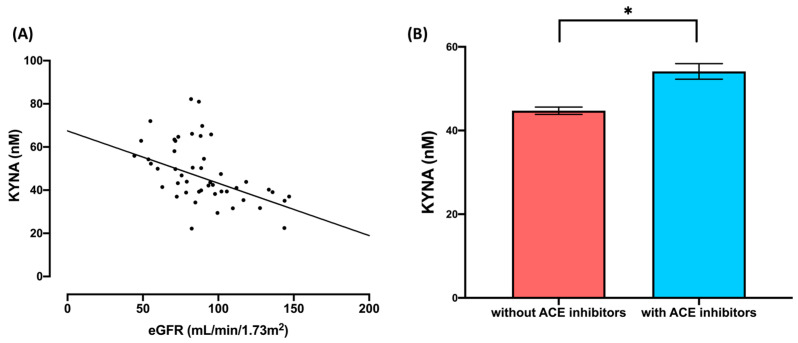
Effects of the eGFR and use of ACE inhibitors on plasma content of kynurenic acid (KYNA): (**A**) correlation between eGFR and kynurenic acid (KYNA); (**B**) plasma content of kynurenic acid (KYNA) in scleroderma patients who are users and non-users of ACE inhibitors; * *p* ≤ 0.05.

Likewise, a MANCOVA was conducted within the patient group to address the secondary aim. The combined dependent variables in the model were TRP, KYN, and KYNA, while the grouping factor was the use of ACE inhibitors (“yes” and “no”). The variable “BMI” was included as a covariate (see Table 4). The results of the MANCOVA multivariate tests revealed a statistically significant effect of ACE inhibitor use on the combined dependent variables in the model (F_3,38_ = 3.57, Wilks’ Lambda = 0.78, *p* = 0.02), as well as on eGFR (F_3,38_ = 4.07, Wilks’ Lambda = 0.76, *p* = 0.01). Furthermore, the univariate tests indicated significant differences in the variable of ACE inhibitor use for KYNA (F_1,40_ = 5.62, *p* = 0.02, η^2^_p_ = 0.12). Additionally, the univariate test revealed significant differences in eGFR and KYNA (F_1,40_ = 6.06, *p* = 0.02, η^2^_p_ = 0.12; Figure 3A). Further details of the MANCOVA results, including the multivariate and univariate tests, can be found in Table 4.

Regarding the MANCOVA results, univariate analysis showed significant results for the factor use of ACE inhibitors and KYNA concentrations. A Bonferroni corrected post hoc test was computed to determine possible differences between subgroups. The latter analysis on KYNA revealed significant differences between SSc patients who use ACE inhibitors and those who do not use ACE inhibitors (M_D_ = 9.30; *p* = 0.02; 95%CI [1.37; 17.19], Figure 3B).

## 3. Discussion

Statistical analysis concerning both male and female SSc patients reveal a decrease in TRP levels and an increase in KYN levels, which is consistent with results published previously [7,24,25,26,27,28,29]. In contrast to Pellicano et al. [30], in our SSc population there was no change in KYNA levels. Furthermore, there was an increase in the KYN/TRP ratio, analogous to that reported by Campochiaro et al., 2019 [26], as well as an increase in KYNA/TRP ratio and a decrease in KYNA/KYN ratio resembling IDO and TDO activity, which has not been previously explored. Generally, our results confirmed the KP dysregulation in SSc. Further sex-stratified analysis of data performed for men and women separately revealed decreased TRP concentrations and increased KYNA/TRP ratio and IDO and TDO in both male and female SSc patients compared to their respective controls. Differently, SSc males but not females exhibited higher KYN concentrations and decreased KYNA/KYN ratio relative to their controls. KYNA content was unaltered in patients of both sexes compared to controls. Regarding our secondary objective, we found that patients with SSc who were taking ACE inhibitors exhibited higher concentrations of KYNA in their serum compared to those who were not taking ACE inhibitors. Additionally, we identified a significant correlation between eGFR and KYNA.

This study concerning the KP, in which data obtained from SSc patients were separated by gender and analyzed independently, is the first of its kind reported in the literature. Since the more severe course of the disease and higher mortality is reported in men than in women [19,20,21], the results of our study allow the formulation of the hypothesis that the difference in KP activity between men and women with SSc is an important factor involved in the disease pathomechanism affecting the course and outcome of SSc. Based on our results, it can be hypothesized that the marked elevation of blood KYN concentration found in men predisposes them to rapid disease progression. Thus, the use of inhibitors of IDO and TDO, enzymes responsible for synthesis of KYN from TRP, in the treatment of SSc can be postulated. This suggestion is attractive because IDO and TDO inhibitors are currently being tested in clinical trials as adjunctive drugs for cancer treatment [31]. On the other hand, it cannot be ruled out that alteration of the KP activity of TRP metabolism has a protective physiological significance in SSc. This idea corresponds to the concept recently presented by Stone and Williams, who postulated that metabolites formed along the KP are mediators of the neuroimmune “reflex” that coordinates protection against stress [32]. Similarly, Jia et al. presented data on the tolerogenic effects of TDO2 and demonstrated that overexpression of TDO2 in dendritic cells ameliorates collagen-induced arthritis severity in mice [33].

Our results evidenced that TRP levels are reduced in both men and women with SSc. Similarly, TRP depletion in SSc patients was reported repeatedly [7,24,25,26,27,29]. It is worth noting that TRP is reduced in newly diagnosed SSc patients, indicating that this process begins early in the disease course [29]. Moreover, TRP depletion was demonstrated in two animal models of fibrosis in mice [29]. Therefore, the role of TRP in SSc should be under debate. TRP is an essential amino acid that is supplied with food. TRP has been recognized as a building block for protein synthesis and as a precursor of serotonin and melatonin, both modulators of high biological significance in the brain and in the periphery. At present, it is widely accepted that approximately 95% of absorbed TRP is metabolized along the KP, which leads mainly to the formation of NAD/NADP(H) and some KP metabolites formed on the side branches of this route [34]. Much less is known about TRP’s ability to act directly on specific molecular targets. Interestingly, it has been shown that TRP is an agonist of two G-protein receptors, GPR139 and GPR142 [35,36]. GPR139 is present mainly in the central nervous system [35] but GPR142 is highly expressed on immune cells and it was found that its receptor antagonist CLP-3094 alleviated collagen antibody-induced arthritis in mice [36]. Whether this receptor is implicated in the process of SSc is currently unknown. It was evidenced that TRP depletion results in increased expression and sensitization of the AhR located on regulatory T lymphocytes [37] considered one of the key players in SSc [38]. On the other hand, it has been proven that TRP depletion inhibits T cells [34,39]. Although it has been indicated for many years that TRP is reduced in SSc patients, its role in the disease induction remains unclear.

According to our results, SSc patients have an increased KYN/TRP ratio indicating increased activity of IDO and TDO. Consequently, an increase of the serum KYN concentration was statistically significant in SSc men but not in SSc women in relation to healthy subjects. KYN is an AhR agonist that is involved in numerous physiological and pathological processes [40] including an impact on the immune system [32,41]. However, the role of this receptor in SSc is poorly understood. Solvay et al., 2023, reported that TRP depletion results in increased expression of AhR and enhanced effects exerted by its agonists [37]. Since in SSc there are a reduced levels of TRP and increased levels of KYN in men, these two phenomena may in concert exacerbate disease pathological processes and result in rapid and more aggressive progress of SSc in men in comparison to women. Contrary to this assumption, Shi et al., 2020, showed that AhR is downregulated in fibroblast obtained from SSc patients. Furthermore, they confirmed an antifibrotic effect of AhR agonism in SSc model in mice [42]. More recently, this same group evidenced that UVA1 irradiation, which is a phototherapy procedure used in local SSc, reduced fibrosis via an AhR mechanism [43]. Interestingly, KYNA is also an AhR agonist [44] with known antifibrotic properties [13]. In our study, there was no change in KYNA levels in both men and women compared to healthy controls. On the other hand, the KYNA/KYN ratio, which is considered as a metabolic indicator of KYN toward KYNA metabolism, was found to be significantly reduced in men with SSc. Furthermore, we found that the KYNA level was elevated in SSc patients treated with ACE inhibitors. This effect can be related to the competition between ACE inhibitors and KYNA for a common organic anion transporter (OAT1/3) in the kidney [45,46].

Since KYNA possesses anti-inflammatory and antioxidant properties [47,48,49,50], its beneficial effect in SSc cannot be ruled out. Thus, further research is needed to reveal whether it plays a role in the course of this disease and treatment effectiveness.

### Study Limitations

Due to the limited number of patients enrolled in the study, the subjects were not divided into subgroups based on the presence of specific antibodies, disease severity, and internal organ involvement. During the collection of blood samples, the patients continued pharmacotherapy. Therefore, the influence of ongoing treatment on the obtained results cannot be ruled out. In fact, higher KYNA levels were found in patients using ACE inhibitors.

Since our analyses were conducted separately for women and separately for men, the effect of sex hormones on TRP metabolism was not studied and discussed in detail. Existing data from the literature indicate that estrogen inhibits KAT activity and increases TRP and quinolinic acid excretion [51,52]. In contrast, progesterone does not affect the excretion of tryptophan metabolites but in high doses it reduces the activation of the KP by IFN-ɣ and decreases quinolinic acid levels, while increasing KYNA levels [53]. On the other hand, SSc alters sex hormone levels [54,55]. Thus, future studies should take into account the endocrine activity of SSc patients, and perhaps the results will give an assumption to include hormone measurement in a panel of basic or complementary tests in SSc patients.

Excretion of TRP, KYN, and KYNA in the urine was not studied in our study. Since previous publications indicate increased urinary excretion of KYN and KYNA in SSc patients [10,11,12], it cannot be excluded that the results of measuring the current level of KP metabolites in the blood do not provide a complete picture of the processes occurring in the affected tissues. Considering that KYN excretion is increased, and yet the KYN level in the blood does not change, as is the case in women with SSc, it can be presumed that KYN production is in fact elevated, at least by an amount to compensate for increased urine excretion of KYN. Similarly, if the excretion of KYNA in the urine increases, and the level of KYNA in the blood does not change, it can indicate that its production is increased. Thus, for a more complete understanding of the fate of TRP and its KP metabolites data on their excretion in SSc patients are needed. Regrettably, there are no data in the literature on the simultaneous measurement of the content of TRP and its metabolites in the blood and urine in the same patient with SSc. Thus, it is legitimate to postulate determination of substances of interest both in blood and urine in the future studies. This conclusion is strengthened by our finding of an inverse relationship between KYNA and eGRF in SSc.

Summing up, in this study we found decreased TRP content and increased KYNA/TRP ratio and KYN/TRP ratio reflecting IDO and TDO activity in both male and female SSc patients. This phenomenon was noted in newly diagnosed SSc patients and did not alter when the treatment was started. Moreover, in SSc males but not females an elevated serum KYN concentrations and decreased KYNA/KYN ratio, an index of KAT activity, was proven. Thus, the obtained data allow us to conclude that the KP is differently dysregulated in men and women with SSc. Although in the current stage of knowledge, the relevance of these changes for the disease process progression cannot be conclusively explained, the results of our study indicate the potential future studies focused on TRP metabolism via the KP in SSc. We propose that more attention should be paid to gender-related differences in SSc and that measurements of KP metabolites should be taken in both blood and urine to determine not only the simple concentrations, but also their turnover. We are also convinced that it is crucial to carry out studies to clarify the role of the receptors which are targeted by KP metabolites because the lack of such data precludes formulation of rational conclusions about the importance of KP metabolites in the course of SSc. Despite many concerns, it is reasonable to postulate inclusion of determination of TRP and KYN metabolites in an extended diagnostic analysis performed at all stages of the SSc, (a) for early diagnosis of the disease, (b) to monitor its course and severity and predict the prognosis, and (c) to assess the efficacy of employed treatment.

## 4. Materials and Methods

### 4.1. Participants and Selection Criteria

The study protocol was approved by the Ethical Committee of the Medical University in Lublin, Poland (No. KE-0254/20/2020). All subjects gave written consent before their enrollment. Analyses were performed on the material obtained from 48 adult SSc patients treated in the Department of Rheumatology and Connective Tissue Diseases, Medical University of Lublin, Poland. The medical examination was performed by experienced rheumatologists. All patients fulfilled the 2013 American College of Rheumatology/European League Against Rheumatism criteria for diagnosis of SSc [3]. The exclusion criteria were as follows: pregnancy or lactation; major surgery (including joint surgery) within 8 weeks prior to the study; rheumatic autoimmune disease other than SSc, including rheumatoid arthritis, systemic lupus erythematosus, mixed connective tissue disease, polymyositis, dermatomyositis, primary Sjögren syndrome; active infection, evidence of malignant disease, or malignancies diagnosed within the previous 5 years; a history of alcohol, drug, or chemical abuse within 1 year prior to the study. A total of 53 healthy controls were recruited. Blood samples were collected in the morning after overnight fasting. Blood was centrifuged and separated serum stored at −80 °C until analysis of KP metabolites. Other blood laboratory measurements were performed as a part of routine clinical practice in certified laboratory. A concise description of the demographic features of the patients and healthy controls is presented in Section 2 and Table 1.

### 4.2. Chromatographic Analysis

Tryptophan (TRP), L-kynurenine sulfate (KYN), and kynurenic acid (KYNA) were obtained from Sigma-Aldrich, Co. (St. Louis, MO, USA) and used as a standard. HPLC-grade chemicals were purchased from J.T. Baker Chemicals (Aventor Performance Materials Poland S.A., Gliwice, Poland) or Sigma-Aldrich. TRP, KYN, and KYNA were analyzed by high-performance liquid chromatography (HPLC), according to the protocol of Zhao et al. (2010) [56], with minor modifications. Briefly, each serum sample was added 6% HClO_4_ and centrifuged at 12,000× *g* for 30 min at 4 °C. The resulting supernatant was applied to an HPLC system consisting of the Dionex P680 Pump, UltiMate 3000 Autosampler with column compartment, RS Variable Wavelength UltiMate 3000 Detector, and RF 2000 Fluorescence Detector (Dionex, Sunnyvale, CA, USA). An Agilent HC-C18 column (250 × 4.6 mm i.d.; 5-µm particle size) was used. The mobile phase was composed of 20 mM sodium acetate, 5 mM zinc acetate, and 4% acetonitrile; the flow rate was 1.0 mL/min. Detectors were set at wavelengths 250 nm for TRP, 365 nm for KYN, and at excitation wavelength of 348 nm and emission at 398 nm for KYNA determination. Control of the HPLC system and data analysis were performed with the Chromeleon software (Dionex^TM^ Chormeleon^TM^ version 7.2.6 (10049), serial number 117836).

### 4.3. Statistical Analysis

Descriptive data are provided in both text and tables to enhance readability. Quantitative variables that approximated a Gaussian distribution are reported as mean ± standard deviation in the text. Non-Gaussian-distributed variables are presented as a median and interquartile range, if necessary. Categorical variables and count data are presented in the text as ratios and percentages. Decimal data are rounded to the nearest decimal place. If the data were <0.00001 or >100,000, we express it in scientific notation. The general information pertaining to the participants is described in the subsequent section and displayed in Table 1. To assess differences between scleroderma patients and healthy controls, the Student *t*-test was employed for Gaussian-distributed data. If necessary, the Mann–Whitney U test was used for initial comparisons between the two groups. For categorical or count data, the χ^2^ test was utilized. In such cases, groups were considered homogeneous if the *p*-value for the χ^2^ test exceeded 0.05.

To compare the means between groups for both the first and second aims, multivariate analyses of covariance (MANCOVA) were conducted. For the first aim, a MANCOVA was performed with TRP, its metabolites (KYN, KYNA), and enzyme activity indicators (KYN/TRP ratio, KYNA/KYN ratio, and KYNA/TRP ratio) as a combined dependent variable; age was included as a covariate and status (i.e., SSc or HC group) was included in the analysis. To investigate and compare sex differences, we performed the same analysis for males and females separately. The second MANCOVA included TRP and its breakdown metabolites (KYN and KYNA) as a combined dependent variable, estimated glomerular filtration rate (eGFR) and body mass index (BMI) as covariates, and the use of ACE inhibitors among SSc patients as the grouping factor. Furthermore, eGFR was calculated using the 2021 adapted version of the CKD-EPI creatinine equation, which adjusts the glomerular filtration rate based on age, sex, and serum creatinine [57].

Mean differences between groups were considered statistically significant if the two-tailed *p*-value was equal or less than 0.05; if the *p*-value was lower than 0.001, we express it as <0.001. For effect sizes, partial eta-square (η^2^_p_) values were calculated. For multivariate tests, the effect size was calculated using the formula: η^2^_p_ = (df_1_ × F) / [(df_1_ × F) + df_2_] [13,14]. In univariate tests, the effect size was calculated as η^2^_p_ = (SS_effect_) / [SS_effect_ + SS_error_] [58,59]. Consistent with established definitions, effect sizes were categorized as follows: very small (η^2^_p_ < 0.01), small (0.01 ≤ η^2^_p_ < 0.06), moderate (0.06 ≤ η^2^_p_ < 0.14), and large (η^2^_p_ ≥ 0.14) [58,59]. Concerning the effect sizes, we rounded to the nearest decimal place, and values lower than 0.01 are expressed as < 0.01.

Finally, statistical analyses were conducted using SPSS version 25.0 (Statistical Package for the Social Sciences, International Business Machines Corporation, Armonk, NY, USA). Model graphs were generated using Prism 8 GraphPad (GraphPad Software Inc., La Jolla, CA, USA).

## Figures and Tables

**Table 1:** General characteristics of scleroderma patients (SSc) and healthy controls (HC): age, sex, plasma content of tryptophan (TRP), kynurenine (KYN), and kynurenic acid (KYNA) and enzyme activity indicators.

	SSc Patients (n = 48)	HC (n = 53)	*t*- or χ^2^-Test	*p* <
Age	51.17 (13.15)	46.77 (13.36)	1.66	0.100
Sex (m/f)	19/29	27/26	1.31	0.252
Plasma content				
[TRP]	29,524.88 (7282.10)	38,284.19 (7240.42)	−6.06	<0.001
[KYN]	4452.07 (1673.82)	3453.23 (1359.69)	3.30	0.001
[KYNA]	47.87 (14.05)	49.00 (15.93)	−0.38	0.706
Enzyme activity *				
IDO and TDO	0.16 (0.06)	0.09 (0.03)	7.28	<0.001
KATs	0.01 (0.004)	0.02 (0.006)	−3.64	<0.001
KYNA/TRP	0.002 (0.0004)	0.0013 (0.0005)	3.93	<0.001

Data are presented as a mean ± SD (in brackets) except for sex; n—number of subjects; * enzyme activity was calculated as a metabolite to precursor ratio, IDO and TDO = [KYN]/[TRP]; KATs = [KYNA/KYN]. Differences between SSc patients and HC were calculated using independent samples *t*-test, with the exception of sex differences (χ^2^-test). Values of all metabolites are in nM (nanomoles). Abbreviations: IDO = indoleamine-2,3-dioxygenase; KATs = kynurenine aminotransferases; KYN = kynurenine; KYNA = kynurenic acid; TDO = tryptophan 2,3-dioxygenase; TRP = tryptophan.

**Table 2:** MANCOVA for male subjects (male SSc and male HC): plasma content of tryptophan (TRP), kynurenine (KYN), and kynurenic acid (KYNA) and enzyme activity indicators, controlling for age (grouping factors: group status and sex).

Multivariate Tests							
		Pillai’s Trace **	F	*df*_1_	*df*_2_	*p*	η^2^_p_
Age		0.134	0.983	6	38	0.450	0.134
Status		0.649	11.701	6	38	* <0.001	0.649
Univariate Tests							
	DV	Type III SS	df	MS	F	*p*	η^2^_p_
Age	[TRP]	6.00 × 10^4^	1	6.00 × 10^4^	0.001	0.975	<0.01
	[KYN]	3.87 × 10^6^	1	3.87 × 10^6^	1.853	0.181	0.04
	[KYNA]	45.60	1	45.60	0.141	0.709	0.003
	IDO and TDO ***	0.01	1	0.01	3.321	0.075	0.07
	KATs ***	5.43 × 10^−5^	1	5.43 × 10^−5^	1.428	0.239	0.03
	[KYNA]/[TRP]	5.49 × 10^−9^	1	5.49 × 10^−9^	0.019	0.892	<0.01
Status	[TRP]	8.36 × 10^8^	1	8.36 × 10^8^	13.715	* 0.001	0.242
	[KYN]	4.10 × 10^7^	1	4.10 × 10^7^	19.637	* <0.001	0.314
	[KYNA]	74.58	1	74.58	0.231	0.633	0.005
	IDO and TDO ***	0.10	1	0.10	50.391	* <0.001	0.540
	KATs ***	3.61 × 10^−4^	1	3.61 × 10^−4^	9.512	* 0.004	0.181
	[KYNA]/[TRP]	1.89 × 10^−6^	1	1.89 × 10^−6^	6.418	* 0.015	0.130
Residuals	[TRP]	2.62 × 10^9^	43	6.10 × 10^7^			
	[KYN]	8.99 × 10^7^	43	2.09 × 10^6^			
	[KYNA]	1.39 × 10^4^	43	322.79			
	IDO and TDO ***	0.08	43	0.002			
	KATs ***	0.002	43	3.799 × 10^−5^			
	[KYNA]/[TRP]	1.267 × 10^−5^	43	2.946 × 10^−7^			

Two-tailed *p* -values (*p* ≤ 0.05) are flagged (*). Values of all metabolites are in nM (nanomoles). ** Since the Box’s M test of equality of covariances was significant (*p* < 0.001), we used Pillai’s trace as test statistic to express the results of the multivariate test. *** Values represent enzyme activity (IDO and TDO = [KYN]/[TRP], KATs = [KYNA]/[KYN]). Abbreviations: F = F-test value; *p* = *p*-value; DV = dependent variable; SS = sum of Squares; df = degrees of freedom; MS = mean square; η^2^_p_ = partial eta-squared (effect size); IDO = indoleamine-2,3-dioxygenase; KATs = kynurenine aminotransferases; KYN = kynurenine; KYNA = kynurenic acid; TDO = tryptophan 2,3-dioxygenase; TRP = tryptophan.

**Table 3:** MANCOVA for female subjects (female SSc and female HC): plasma content of tryptophan (TRP), kynurenine (KYN), and kynurenic acid (KYNA) and enzyme activity indicator, controlling for age (grouping factors: group status and sex).

Multivariate Tests							
		Pillai’s Trace **	F	*df*_1_	*df*_2_	*p*	η^2^_p_
Age		0.175	1.660	6.000	47.000	0.152	0.175
Status		0.421	5.707	6.000	47.000	* <0.001	0.421
Univariate Tests							
	DV	Type III SS	df	MS	F	*p*	η^2^_p_
Age	[TRP]	4.60 × 10^5^	1	4.60 × 10^5^	0.010	0.922	<0.01
	[KYN]	2.01 × 10^6^	1	2.01 × 10^6^	1.042	0.312	0.02
	[KYNA]	287.36	1	287.36	2.157	0.148	0.04
	IDO and TDO ***	0.002	1	0.002	1.542	0.220	0.03
	KATs ***	1.51 × 10^−7^	1	1.51 × 10^−7^	0.008	0.928	<0.01
	[KYNA]/[TRP]	5.19 × 10^−7^	1	5.19 × 10^−7^	6.076	0.017	0.11
Status	[TRP]	9.20 × 10^8^	1	9.20 × 10^8^	19.414	* <0.001	0.272
	[KYN]	8.65 × 10^5^	1	8.65 × 10^5^	0.447	0.507	0.009
	[KYNA]	129.68	1	129.68	0.974	0.328	0.018
	IDO and TDO ***	0.02	1	0.02	14.284	* <0.001	0.215
	KATs ***	5.17 × 10^−5^	1	5.17 × 10^−5^	2.816	0.099	0.051
	[KYNA]/[TRP]	9.75 × 10^−7^	1	9.75 × 10^−7^	11.420	* 0.001	0.180
Residuals	[TRP]	2.46 × 10^9^	52	4.74 × 10^7^			
	[KYN]	1.01 × 10^8^	52	1.93 × 10^6^			
	[KYNA]	6926.54	52	133.20			
	IDO and TDO ***	0.08	52	0.002			
	KATs ***	0.001	52	1.84 × 10^−5^			
	[KYNA]/[TRP]	4.44 × 10^−6^	52	8.54 × 10^−8^			

Two-tailed *p*-values (*p* ≤ 0.05) are flagged (*). Values of all metabolites are in nM (nanomoles). ** Since the Box’s M test of equality of covariances was significant (*p* < 0.001), we used Pillai’s trace as test statistics to express the results of the multivariate test. *** Values represent enzyme activity (IDO and TDO = [KYN]/[TRP], KATs = [KYNA]/[KYN]). Abbreviations: F = F-test value; *p* = *p*-value; DV = dependent variable; SS = sum of Squares; df = degrees of freedom; MS = mean square; η^2^_p_ = partial eta-squared (effect size); IDO = indoleamine-2,3-dioxygenase; KATs = kynurenine aminotransferases; KYN = kynurenine; KYNA = kynurenic acid; TDO = tryptophan 2,3-dioxygenase; TRP = tryptophan.

**Table 4:** MANCOVA: plasma content of tryptophan (TRP), kynurenine (KYN), and kynurenic acid (KYNA), controlling for eGFR and BMI (grouping factor: use of ACE inhibitors).

Multivariate Tests							
		Wilks’ Lambda **	F	*df*_1_	*df*_2_	*p*	η^2^_p_
eGFR		0.76	4.07	3	38	* 0.01	0.24
BMI		0.98	0.29	3	38	0.83	0.02
Use of ACEi		0.78	3.57	3	38	* 0.02	0.22
Univariate Tests							
	DV	Type III SS	df	MS	F	*p*	η^2^_p_
eGFR ***	[TRP]	3.32 × 10^6^	1	3.32 × 10^6^	0.07	0.80	<0.01
	[KYN]	8.81 × 10^6^	1	8.81 × 10^6^	3.03	0.09	0.07
	[KYNA]	822.95	1	822.95	6.06	* 0.02	0.13
BMI	[TRP]	1.39 × 10^6^	1	1.39 × 10^6^	0.03	0.87	<0.01
	[KYN]	1.41 × 10^6^	1	1.41 × 10^6^	0.48	0.49	0.01
	[KYNA]	0.04	1	0.04	0.00	0.99	<0.01
Use of ACEi	[TRP]	4.75 × 10^7^	1	4.75 × 10^7^	0.97	0.33	0.02
	[KYN]	19,159.33	1	19,159.33	0.01	0.94	0.00
	[KYNA]	762.73	1	762.73	5.62	* 0.02	0.12
Residuals	[TRP]	1.95 × 10^9^	40	4.87 × 10^7^			
	[KYN]	1.16 × 10^8^	40	2.91 × 10^6^			
	[KYNA]	5430.12	40	135.75			

Two-tailed *p*-values (*p* ≤ 0.05) are flagged (*). Values of all metabolites are in nM (nanomoles). ** Since the Box’s M test of equality of covariances was not significant (*p* = 0.05), we used Wilks’ Lambda as test statistics to express the results of the multivariate test. *** eGFR calculated using the 2021 adapted version of CKD-EPI (Chronic Kidney Disease Epidemiology Collaboration) equation, adjusted for sex, age, and serum creatinine (The National Kidney Foundation: https://www.kidney.org/professionals/kdoqi/gfr_calculator/formula; accessed on 16 July 2023). Abbreviations: F = F-test value; *p* = *p*-value; DV = dependent variable; SS = sum of Squares; df = degrees of freedom; MS = mean square; η^2^_p_ = partial eta-squared (effect size); ACEi = angiotensin-converting-enzyme inhibitors; BMI = body-mass-index; eGFR = glomerular filtration rate; KYN = kynurenine; KYNA = kynurenic acid; TRP = tryptophan.

## Data Availability

All data generated or analyzed during this study are included in this published article.

## References

[1] Bairkdar M., Rossides M., Westerlind H., Hesselstrand R., Arkema E.V., Holmqvist M. (2021). Incidence and Prevalence of Systemic Sclerosis Globally: A Comprehensive Systematic Review and Meta-Analysis. Rheumatology.

[2] Allanore Y., Simms R., Distler O., Trojanowska M., Pope J., Denton C.P., Varga J. (2015). Systemic Sclerosis. Nat. Rev. Dis. Primers.

[3] van den Hoogen F., Khanna D., Fransen J., Johnson S.R., Baron M., Tyndall A., Matucci-Cerinic M., Naden R.P., Medsger T.A., Carreira P.E. (2013). 2013 Classification Criteria for Systemic Sclerosis: An American College of Rheumatology/European League against Rheumatism Collaborative Initiative. Ann. Rheum. Dis..

[4] Savitz J. (2020). The Kynurenine Pathway: A Finger in Every Pie. Mol. Psychiatry.

[5] Simpson C.E., Ambade A.S., Harlan R., Roux A., Aja S., Graham D., Shah A.A., Hummers L.K., Hemnes A.R., Leopold J.A. (2023). Kynurenine Pathway Metabolism Evolves with Development of Preclinical and Scleroderma-Associated Pulmonary Arterial Hypertension. Am. J. Physiol.—Lung Cell Mol. Physiol..

[6] Morales-González V., Galeano-Sánchez D., Covaleda-Vargas J.E., Rodriguez Y., Monsalve D.M., Pardo-Rodriguez D., Cala M.P., Acosta-Ampudia Y., Ramírez-Santana C. (2023). Metabolic Fingerprinting of Systemic Sclerosis: A Systematic Review. Front. Mol. Biosci..

[7] Bögl T., Mlynek F., Himmelsbach M., Sepp N., Buchberger W., Geroldinger-Simić M. (2022). Plasma Metabolomic Profiling Reveals Four Possibly Disrupted Mechanisms in Systemic Sclerosis. Biomedicines.

[8] Rodgers J., Stone T.W., Barrett M.P., Bradley B., Kennedy P.G.E. (2009). Kynurenine Pathway Inhibition Reduces Central Nervous System Inflammation in a Model of Human African Trypanosomiasis. Brain.

[9] Houpt J.B., Ogryzlo M.A., Hunt M. (1973). Tryptophan Metabolism in Man (with Special Reference to Rheumatoid Arthritis and Scleroderma). Semin. Arthritis Rheum..

[10] Pinals R.S. (1964). Tryptophan Metabolism in Rheumatic Disease. Arthritis Rheum..

[11] Binazzi M., Calandra P. (1973). Tryptophan to Niacin Pathway in Scleroderma and in Dermatomyositis. Arch. Dermatol. Forsch..

[12] De Antoni A., Muggeo M., Costa C., Allegri G., Crepaldi G. (1976). Tryptophan Metabolism “via” Nicotimic Acid in Patients with Scleroderma. Acta Vitaminol. Enzym..

[13] Poormasjedi-Meibod M.-S., Hartwell R., Kilani R.T., Ghahary A. (2014). Anti-Scarring Properties of Different Tryptophan Derivatives. PLoS ONE.

[14] Li Y., Kilani R.T., Rahmani-Neishaboor E., Jalili R.B., Ghahary A. (2014). Kynurenine Increases Matrix Metalloproteinase-1 and -3 Expression in Cultured Dermal Fibroblasts and Improves Scarring in Vivo. J. Investig. Dermatol..

[15] Nestor M.S., Berman B., Fischer D.L., Han H., Gade A., Arnold D., Lawson A. (2021). A Randomized, Double-Blind, Active- and Placebo-Controlled Trial Evaluating a Novel Topical Treatment for Keloid Scars. J. Drugs Dermatol..

[16] Papp A., Hartwell R., Evans M., Ghahary A. (2018). The Safety and Tolerability of Topically Delivered Kynurenic Acid in Humans. A Phase 1 Randomized Double-Blind Clinical Trial. J. Pharm. Sci..

[17] Sagonas I., Daoussis D. (2022). Serotonin and Systemic Sclerosis. An Emerging Player in Pathogenesis. Jt. Bone Spine.

[18] Peoples C., Medsger T.A., Lucas M., Rosario B.L., Feghali-Bostwick C.A. (2016). Gender Differences in Systemic Sclerosis: Relationship to Clinical Features, Serologic Status and Outcomes. J. Scleroderma Relat. Disord..

[19] Hughes M., Pauling J.D., Armstrong-James L., Denton C.P., Galdas P., Flurey C. (2020). Gender-Related Differences in Systemic Sclerosis. Autoimmun. Rev..

[20] Liem S.I.E., Ciaffi J., Van Leeuwen N.M., Boonstra M., Ahmed S., Beaart-van De Voorde L.J.J., Corsel A., Dhondai T., Ninaber M.K., Geelhoed-Veltman J.J.M. (2023). Step Forward in Early Recognition of Systemic Sclerosis: Data from the Leiden CCISS Cohort. RMD Open.

[21] De Angelis R., Giuggioli D., Bajocchi G., Dagna L., Zanframundo G., Foti R., Cacciapaglia F., Cuomo G., Ariani A., Rosato E. (2022). Sex-Related Differences in Systemic Sclerosis: A Multicenter Cross-Sectional Study From the National Registry of the Italian Society for Rheumatology. J. Rheumatol..

[22] Volkmann E.R., Andréasson K., Smith V. (2023). Systemic Sclerosis. Lancet.

[23] Hughes M., Herrick A.L. (2019). Systemic Sclerosis. Br. J. Hosp. Med..

[24] Csipô I., Czirják L., Szántó S., Szerafin L., Sipka S., Szegedi G. (1995). Decreased Serum Tryptophan and Elevated Neopterin Levels in Systemic Sclerosis. Clin. Exp. Rheumatol..

[25] Bengtsson A.A., Trygg J., Wuttge D.M., Sturfelt G., Theander E., Donten M., Moritz T., Sennbro C.-J., Torell F., Lood C. (2016). Metabolic Profiling of Systemic Lupus Erythematosus and Comparison with Primary Sjögren’s Syndrome and Systemic Sclerosis. PLoS ONE.

[26] Campochiaro C., Lytton S., Nihtyanova S., Fuchs D., Ong V.H., Denton C.P. (2019). Elevated Kynurenine Levels in Diffuse Cutaneous and Anti-RNA Polymerase III Positive Systemic Sclerosis. Clin. Immunol..

[27] Meier C., Freiburghaus K., Bovet C., Schniering J., Allanore Y., Distler O., Nakas C., Maurer B. (2020). Serum Metabolites as Biomarkers in Systemic Sclerosis-Associated Interstitial Lung Disease. Sci. Rep..

[28] Smolenska Z., Zabielska-Kaczorowska M., Wojteczek A., Kutryb-Zajac B., Zdrojewski Z. (2020). Metabolic Pattern of Systemic Sclerosis: Association of Changes in Plasma Concentrations of Amino Acid-Related Compounds with Disease Presentation. Front. Mol. Biosci..

[29] Guo M., Liu D., Jiang Y., Chen W., Zhao L., Bao D., Li Y., Distler J.H.W., Zhu H. (2023). Serum Metabolomic Profiling Reveals Potential Biomarkers in Systemic Sclerosis. Metabolism.

[30] Pellicano C., Vaiarello V., Colalillo A., Gigante A., Iannazzo F., Rosato E. (2022). Role of Kinurenic Acid in the Systemic Sclerosis Renal Involvement. Clin. Exp. Med..

[31] Amobi A., Qian F., Lugade A.A., Odunsi K., Kalinski P. (2017). Tryptophan Catabolism and Cancer Immunotherapy Targeting IDO Mediated Immune Suppression. Tumor Immune Microenvironment in Cancer Progression and Cancer Therapy.

[32] Stone T.W., Williams R.O. (2023). Modulation of T Cells by Tryptophan Metabolites in the Kynurenine Pathway. Trends Pharmacol. Sci..

[33] Jia C., Wang Y., Wang Y., Cheng M., Dong W., Wei W., Zhao Y., Chang Y. (2024). TDO2-Overexpressed Dendritic Cells Possess Tolerogenicity and Ameliorate Collagen-Induced Arthritis by Modulating the Th17/Regulatory T Cell Balance. J. Immunol..

[34] Klaessens S., Stroobant V., De Plaen E., Van den Eynde B.J. (2022). Systemic Tryptophan Homeostasis. Front. Mol. Biosci..

[35] Liu C., Bonaventure P., Lee G., Nepomuceno D., Kuei C., Wu J., Li Q., Joseph V., Sutton S.W., Eckert W. (2015). GPR139, an Orphan Receptor Highly Enriched in the Habenula and Septum, Is Activated by the Essential Amino Acids l-Tryptophan and l-Phenylalanine. Mol. Pharmacol..

[36] Murakoshi M., Kuwabara H., Nagasaki M., Xiong Y.M., Reagan J.D., Maeda H., Nara F. (2017). Discovery and Pharmacological Effects of a Novel GPR142 Antagonist. J. Recept. Signal Transduct..

[37] Solvay M., Holfelder P., Klaessens S., Pilotte L., Stroobant V., Lamy J., Naulaerts S., Spillier Q., Frédérick R., De Plaen E. (2023). Tryptophan Depletion Sensitizes the AHR Pathway by Increasing AHR Expression and GCN2/LAT1-Mediated Kynurenine Uptake, and Potentiates Induction of Regulatory T Lymphocytes. J. Immunother. Cancer.

[38] Jin W., Zheng Y., Zhu P. (2022). T Cell Abnormalities in Systemic Sclerosis. Autoimmun. Rev..

[39] Mellor A.L., Munn D.H. (1999). Tryptophan Catabolism and T-Cell Tolerance: Immunosuppression by Starvation?. Immunol. Today.

[40] Opitz C.A., Litzenburger U.M., Sahm F., Ott M., Tritschler I., Trump S., Schumacher T., Jestaedt L., Schrenk D., Weller M. (2011). An Endogenous Tumour-Promoting Ligand of the Human Aryl Hydrocarbon Receptor. Nature.

[41] Seo S.-K., Kwon B. (2023). Immune Regulation through Tryptophan Metabolism. Exp. Mol. Med..

[42] Shi Y., Tang B., Yu J., Luo Y., Xiao Y., Pi Z., Tang R., Wang Y., Kanekura T., Zeng Z. (2020). Aryl Hydrocarbon Receptor Signaling Activation in Systemic Sclerosis Attenuates Collagen Production and Is a Potential Antifibrotic Target. Int. Immunopharmacol..

[43] Shi Y., Xiao Y., Yu J., Liu J., Liu L., Ding Y., Qiu X., Zhan Y., Tang R., Zeng Z. (2023). UVA1 Irradiation Attenuates Collagen Production via Ficz/AhR/MAPK Signaling Activation in Scleroderma. Int. Immunopharmacol..

[44] DiNatale B.C., Murray I.A., Schroeder J.C., Flaveny C.A., Lahoti T.S., Laurenzana E.M., Omiecinski C.J., Perdew G.H. (2010). Kynurenic Acid Is a Potent Endogenous Aryl Hydrocarbon Receptor Ligand That Synergistically Induces Interleukin-6 in the Presence of Inflammatory Signaling. Toxicol. Sci..

[45] Burckhardt G. (2012). Drug Transport by Organic Anion Transporters (OATs). Pharm. Ther..

[46] Wu W., Bush K.T., Nigam S.K. (2017). Key Role for the Organic Anion Transporters, OAT1 and OAT3, in the in Vivo Handling of Uremic Toxins and Solutes. Sci. Rep..

[47] Kaszaki J., Palásthy Z., Erczes D., Rácz A., Torday C., Varga G., Vécsei L., Boros M. (2008). Kynurenic Acid Inhibits Intestinal Hypermotility and Xanthine Oxidase Activity during Experimental Colon Obstruction in Dogs. Neurogastroenterol. Motil..

[48] Varga G., Erces D., Fazekas B., Fülöp M., Kovács T., Kaszaki J., Fülöp F., Vécsei L., Boros M. (2010). N-Methyl-D-Aspartate Receptor Antagonism Decreases Motility and Inflammatory Activation in the Early Phase of Acute Experimental Colitis in the Rat. Neurogastroenterol. Motil..

[49] Ramírez Ortega D., Ugalde Muñiz P.E., Blanco Ayala T., Vázquez Cervantes G.I., Lugo Huitrón R., Pineda B., González Esquivel D.F., Pérez de la Cruz G., Pedraza Chaverrí J., Sánchez Chapul L. (2021). On the Antioxidant Properties of L-Kynurenine: An Efficient ROS Scavenger and Enhancer of Rat Brain Antioxidant Defense. Antioxidants.

[50] Lugo-Huitrón R., Blanco-Ayala T., Ugalde-Muñiz P., Carrillo-Mora P., Pedraza-Chaverrí J., Silva-Adaya D., Maldonado P.D., Torres I., Pinzón E., Ortiz-Islas E. (2011). On the Antioxidant Properties of Kynurenic Acid: Free Radical Scavenging Activity and Inhibition of Oxidative Stress. Neurotoxicol. Teratol..

[51] Jayawickrama G.S., Nematollahi A., Sun G., Gorrell M.D., Church W.B. (2017). Inhibition of Human Kynurenine Aminotransferase Isozymes by Estrogen and Its Derivatives. Sci. Rep..

[52] Mason M., Gullekson E.H. (1960). Estrogen-Enzyme Interactions: Inhibition and Protection of Kynurenine Transaminase by the Sulfate Esters of Diethylstilbestrol, Estradiol, and Estrone. JBC.

[53] De Bie J., Lim C.K., Guillemin G.J. (2016). Progesterone Alters Kynurenine Pathway Activation in IFN-γ-Activated Macrophages—Relevance for Neuroinflammatory Diseases. Int. J. Tryptophan Res..

[54] Ciaffi J., Morabito M.F., Ruscitti P., D’Angelo S., Mancarella L., Brusi V., Abignano G., Pucino V., Giacomelli R., Meliconi R. (2021). Incidence, Prevalence and Mortality of Systemic Sclerosis in Italy: A Nationwide Population-Based Study Using Administrative Health Data. Rheumatol. Int..

[55] Jutiviboonsuk A., Salang L., Eamudomkarn N., Mahakkanukrauh A., Suwannaroj S., Foocharoen C. (2021). Prevalence and Clinical Associations with Premature Ovarian Insufficiency, Early Menopause, and Low Ovarian Reserve in Systemic Sclerosis. Clin. Rheumatol..

[56] Zhao J., Gao P., Zhu D. (2010). Optimization of Zn2+-Containing Mobile Phase for Simultaneous Determination of Kynurenine, Kynurenic Acid and Tryptophan in Human Plasma by High Performance Liquid Chromatography. J. Chromatogr. B Anal. Technol. Biomed. Life Sci..

[57] Inker L.A., Eneanya N.D., Coresh J., Tighiouart H., Wang D., Sang Y., Crews D.C., Doria A., Estrella M.M., Froissart M. (2021). New Creatinine- and Cystatin C–Based Equations to Estimate GFR without Race. N. Engl. J. Med..

[58] Morris P.E., Fritz C.O. (2013). Effect Sizes in Memory Research. Memory.

[59] University of Cambridge Rules of Thumb on Magnitudes of Effect Sizes. http://imaging.mrc-cbu.cam.ac.uk/statswiki/FAQ/effectSize.

